# Bright-blood and dark-blood phase sensitive inversion recovery late gadolinium enhancement and T1 and T2 maps in a single free-breathing scan: an all-in-one approach

**DOI:** 10.1186/s12968-021-00823-3

**Published:** 2021-11-08

**Authors:** Peter Kellman, Hui Xue, Kelvin Chow, James Howard, Liza Chacko, Graham Cole, Marianna Fontana

**Affiliations:** 1 National Heart, Lung, and Blood Institute, National Institute of Health, Bethesda, MD USA; 2 Cardiovascular MR R&D, Siemens Medical Solutions USA, Inc., Chicago, IL USA; 3 Imperial College Healthcare NHS Trust, London, UK; 4 National Heart and Lung Institute, Imperial College London, London, UK; 5 Royal Free London NHS Foundation Trust, London, UK; 6 National Amyloidosis Centre, Division of Medicine, University College London, London, UK

**Keywords:** PSIR LGE, T1 map, T2 map, SASHA, Dark-blood LGE

## Abstract

**Background:**

Quantitative cardiovascular magnetic resonance (CMR) T1 and T2 mapping are used to detect diffuse disease such as myocardial fibrosis or edema. However, post gadolinium contrast mapping often lacks visual contrast needed for assessment of focal scar. On the other hand, late gadolinium enhancement (LGE) CMR which nulls the normal myocardium has excellent contrast between focal scar and normal myocardium but has poor ability to detect global disease. The objective of this work is to provide a calculated bright-blood (BB) and dark-blood (DB) LGE based on simultaneous acquisition of T1 and T2 maps, so that both diffuse and focal disease may be assessed within a single multi-parametric acquisition.

**Methods:**

The prototype saturation recovery-based SASHA T1 mapping may be modified to jointly calculate T1 and T2 maps (known as multi-parametric SASHA) by acquiring additional saturation recovery (SR) images with both SR and T2 preparations. The synthetic BB phase sensitive inversion recovery (PSIR) LGE may be calculated from the post-contrast T1, and the DB PSIR LGE may be calculated from the post-contrast joint T1 and T2 maps. Multi-parametric SASHA maps were acquired free-breathing (45 heartbeats). Protocols were designed to use the same spatial resolution and achieve similar signal-to-noise ratio (SNR) as conventional motion corrected (MOCO) PSIR. The calculated BB and DB LGE were compared with separate free breathing (FB) BB and DB MOCO PSIR acquisitions requiring 16 and 32 heart beats, respectively. One slice with myocardial infarction (MI) was acquired with all protocols within 4 min.

**Results:**

Multiparametric T1 and T2 maps and calculated BB and DB PSIR LGE images were acquired for patients with subendocardial chronic MI (n = 10), acute MI (n = 3), and myocarditis (n = 1). The contrast-to-noise (CNR) between scar (MI and myocarditis) and remote was 26.6 ± 7.7 and 20.2 ± 7.4 for BB and DB PSIR LGE, and 31.3 ± 10.6 and 21.8 ± 7.6 for calculated BB and DB PSIR LGE, respectively. The CNR between scar and the left ventricualr blood pool was 5.2 ± 6.5 and 29.7 ± 9.4 for conventional BB and DB PSIR LGE, and 6.5 ± 6.0 and 38.6 ± 11.6 for calculated BB and DB PSIR LGE, respectively.

**Conclusions:**

A single free-breathing acquisition using multi-parametric SASHA provides T1 and T2 maps and calculated BB and DB PSIR LGE images for comprehensive tissue characterization.

## Background

Cardiovascular magnetic resonance (CMR) quantitative T1 and T2 mapping [1–4] are used to detect diffuse disease such as myocardial fibrosis or edema [5–7]. However, post gadolinium contrast mapping often lacks visual contrast needed for assessment of focal scar. On the other hand, late gadolinium enhancement (LGE) which nulls the normal myocardium has excellent contrast between focal scar and normal myocardium but has poor ability to detect global disease. To comprehensively assess both focal and globally diseased myocardium, multiple scans are required, which often requires repeated breath-holding and thereby lengthens the CMR exam. Clinical applicability could be greatly improved if both LGE and tissue relaxation maps can be acquired in a single free-breathing scan. Synthetic LGE images [8, 9] have been calculated from post-contrast T1 maps with excellent agreement with conventional LGE. This provides a significant time savings in cases where both LGE and T1 maps are desired.

A large fraction of myocardial infarctions (MI) caused by coronary artery disease result in subendocardial scar adjacent to the blood pool. Although conventional bright-blood (BB) LGE achieves excellent contrast between infarcted and normal myocardium, the contrast between the scar and the blood pool is frequently poor. This can make it difficult to delineate the subendocardial border when assessing its extent and, in some cases, leads to missing detection entirely.

Dark-blood (DB) LGE CMR is an emerging technique to improve the contrast between sub-endocardial LGE and adjacent blood pool, by combining inversion recovery with either T2 preparation [10, 11] or magnetization transfer [12]. A free-breathing DB phase sensitive inversion recovery (PSIR) LGE approach [11] achieves high quality DB LGE images by combining inversion recovery (IR) with a T2 preparation. In this approach, the T2 weighting suppresses the myocardial signal, thereby shifting the null time of the myocardium with respect to the blood. DB LGE may be used to provide greater contrast between the MI and adjacent blood pool thereby increasing the detectability of MI and improving the confidence of assessment [11, 13]. However, current dark-blood LGE also requires separate acquisition and therefore its integration into routine CMR clinical workflow is limited.

In this paper, we propose a solution to achieve the ideal “all-in-one” imaging for four imaging applications – BB LGE, DB LGE, T1 and T2 mapping, without breath-holding. With this technique, both diffuse and focal disease may be assessed within a single free-breathing (FB) multi-parametric acquisition. In this study, we focus on the extension of mapping to provide LGE images.

A number of recent developments has enabled the simultaneous acquisition of T1 and T2 maps [14–17]. We have chosen the saturation recovery (SR) multi-parametric SASHA method [14], as it may be implemented in a free-breathing protocol that can achieve the desired image quality in a reasonable acquisition time. The synthetic PSIR approach was extended to simultaneously calculate both the bright-blood and dark-blood PSIR LGE from jointly acquired T1 and T2 maps.

This approach provides an overall time savings in the study and produces T1 and T2 maps and LGE images that are spatially co-registered thereby simplifying multi-parametric analysis. The performance and image quality of the proposed approach is compared to individual BB and DB PSIR LGE acquisitions in patients with subendocardial chronic MI and acute MI.

## Methods

**Fig. 1:**
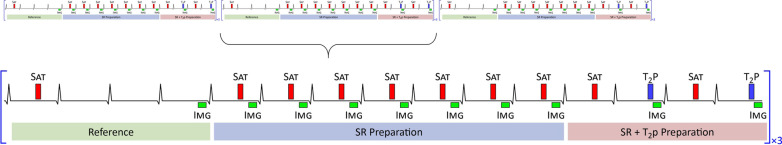
Sequence diagram for multi-parametric saturation recovery single shot acquisition (SASHA) combined T1 and T2 mapping using free-breathing protocol

The prototype saturation recovery-based SASHA [4] T1 mapping may be modified to jointly calculate T1 and T2 maps (known as multi-parametric SASHA or mSASHA) by acquiring additional saturation recovery (SR) images with both SR and T2 preparations [14]. We have adapted the breath-held protocol [18] which had 10 measurements to have 3 repetitions or 30 measurements, to improve the overall precision. The images are acquired FB and non-rigid image registration is used to correct respiratory motion. The multi-parameter sequence is diagrammed in Fig. 1. A series of single shot images are acquired which include reference images with long saturation delay, saturation prepared images, and images with both saturation recovery and T2 preparations. From these images it is possible to jointly fit for pixel wise T1 and T2 maps. Joint fitting has numerous benefits [18] in that the T1 is no longer dependent on T2 and T2 is no longer dependent on T1. Phantom validation of T1 and T2 measurement accuracy using the T1MES phantom [19] was performed using long scans with TR = 10 s as the reference standard. The synthetic BB PSIR LGE may be calculated from the post-contrast T1, and the DB PSIR LGE may be calculated from the joint T1 and T2 maps, as described below.

### Calculated PSIR LGE

The BB-PSIR signal using the standard inversion recovery (IR) LGE sequence is exponentially weighted and may be written as:

1 $$BB \, PSIR \, = {\text{ }}1 - 2e^{{\left( { - TI/T1} \right)}}$$

where the inversion time (TI) is set to null normal myocardium and T1 is the tissue T1. The DB-PSIR LGE signal using an IR-T2 sequence may be written as:

2 $$DB{\mkern 1mu} PSIR{\mkern 1mu} = 1 - {\mkern 1mu} \left[ {1 - \left( {1 - 2e^{{\left( { - TD1/T1} \right)}} } \right)e^{{\left( { - TE/T2} \right)}} } \right]e^{{\left( { - TD2/T1} \right)}}$$

with parameters TD1, TD2, and TE are set following [11] to jointly null the normal myocardium while suppressing the blood and minimizing the T2 preparation echo time TE, and T1 and T2 are the tissue T1 and T2, respectively. In the IR-T2 sequence TD1 and TD2 are the inversion delays before and after the T2 preparation and TE is the echo time of the T2 preparation that determines the degree of T2 weighting. These are diagrammed in Fig. 1b of reference [11].

Although it is possible to directly calculate the BB and DB PSIR signals analogous to exponential signal recovery using Eqs. (1 and 2), we have chosen an alternative formulation which produces a signal that is linearly related to the contrast concentration:

3 $$BB \, PSIR \, = \, R1 - R1m$$

where R1 = 1/T1, and R1m corresponds to the value of R1 for normal myocardium. The BB PSIR calculated in this way nulls the normal myocardium (i.e., R1–R1m = 0 for R1 = R1m) and provides positive contrast, i.e., shorter T1 tissue such as MI will appear brighter (R1 > R1m). In this formulation, the signal intensity in the BB PSIR is directly proportional to R1 which is proportional to the gadolinium concentration, [Gd], since R1 = R1_0_ + r1 [Gd], where R1_0_ is the native R1 prior to administering gadolinium and r1 is the relaxivity of the gadolinium contrast agent assuming fast signal exchange.

Similarly, the dark blood PSIR signal may be formulated to null the normal myocardium subject to fixing the blood at a specified level as:

4 $$DB \, PSIR \, = \, R1 - \frac{R1m}{{\left( {a_{DB} \frac{R2}{{R2m}} + \left( {1 - a_{DB} } \right)} \right)}}$$

with:

5 $$a_{DB} \, = \, \frac{{\frac{R1m \times R2m}{{R1b - LGEb}} - R2m}}{{\left( {R2b - R2m} \right)}}$$

where R1b is R1 for blood, R2 = 1/T2, and R2b and R2m are the R2 for blood and normal myocardium respectively. The DB signal in Eq. (4) is formulated such that DB PSIR signal is the same as the BB PSIR signal [Eq. (3)] in the normal myocardium, i.e., R2 = R2m. LGEb is an adjustable parameter that may be varied to change the degree of blood suppression. The exact value selected is somewhat arbitrary, and may be adjusted to set the blood suppression in a manner similar to the free parameter delta ([11], Fig. 3) as illustrated in the results. It is set to -0.5 Hz in this study. The constant a_DB_ can be solved for analytically as in Eq. (5) which is derived by solving for a_DB_ in Eq. (4) with the value of R1 set equal to the value for blood (R1b) and the value of R2 set equal to the value for blood (R2b) and setting DB PSIR = -0.5. In this formulation, the myocardial signal intensity will again be directly proportional to R1 with parameters set to null the normal myocardium, and to set the blood below the myocardium to a fixed level.

The formulation described by Eqs. (3 and 4) were implemented for this work so that the calculated LGE signal intensity was directly proportional to the contrast concentration.

### Imaging sequences

The new method of calculating synthetic PSIR LGE from T1 and T2 maps was compared to the individual sequences for BB and DB PSIR LGE. T1 and T2 maps were acquired using mSASHA. Protocols were designed to use the same spatial resolution and achieve similar signal-to-noise ratio (SNR) between the calculated and conventional sequences. All sequences were acquired free-breathing using single shot readouts with multiple measurements and used respiratory motion correction (MOCO) averaging [20, 21] to improve image SNR. The calculated PSIR LGE was compared with separate FB BB and DB MOCO PSIR acquisitions requiring 16 and 32 heart beats, respectively [11, 22]. The DB PSIR MOCO protocol used 16 measurements which is 2 × that used in BB PSIR MOCO to offset loss in myocardial SNR due to the T2 weighting [11].

The mSASHA sequence consisted of 30 images acquired over 45 heart beats in order to achieve comparable contrast-to-noise (CNR) with the DB PSIR MOCO. An initial pilot study was performed to optimize protocol parameters for mSASHA, consisting of measuring the raw image SNR and calculating the precision of T1 and T2 for various protocols in order to achieve a performance comparable to the DB PSIR MOCO. The mSASHA protocol used a variable flip angle excitation [23] in order to minimize off-resonance artifacts and to mitigate bias due to T1 recovery. The mSASHA protocol was optimized for post-contrast using saturation recovery time (TS) = 300 ms, with 3 anchor images with long recovery, 6 T2-prepared images with a fixed TE = 55 ms and 1 recovery heartbeat, and 21 SR images requiring 45 heartbeats in total. Anchor images had 3 recovery heart beats following saturation, i.e. recovery duration of 3 RR + TS. The mSASHA incorporated acquisition of additional k-space lines at high flip angle to generate additional high-contrast images [24] used to improve the performance of respiratory motion correction. The SR preparation used a pulse sequel design [25], and an adiabatic T2 preparation was used with fixed echo time. The used of a fixed saturation delay rather than distributed delays has been previously shown to optimize accuracy [26]. Typical parameters for these protocols are listed in Table 1.

**Table 1:** Typical imaging parameters for various PSIR LGE MOCO protocols

	Bright Blood (BB)	Dark Blood (DB)	mSASHA (joint T1 and T2 map)
Preparation	Inversion Preparation	Inversion Preparation & T2 preparation	Saturation Preparation & T2 preparations (see Fig. 1)
Readout (single shot)	SSFP FA_IR_ = 50° FA_PD_ = 8°	SSFP FA_IR_ = 50° FA_PD_ = 8°	SSFP Variable FA (max 100°)
Typical FOV / resolution	360 × 270 mm^2^ 1.4 × 1.9 × 8 mm^3^		
Matrix size	256 × 144 (parallel imaging factor 2 & PF factor 7/8)		
Number of acquired images	8 (16 beats)	16 (32 beats)	30 (45 heart beats)
T2 prep TE	n/a	Variable (10—40 ms)	55 ms
TE/TR	1.2/2.8 ms	1.2/2.8 ms	1.3/2.9 ms

* FA*, flip angle; *FOV*, field-of-view; *TE*, echo time; *TR*, repetition time

### Image reconstruction

The image reconstruction, motion correction, mapping, and calculation of PSIR LGE were performed in-line using the Gadgetron software framework [27]. MOCO incorporated a high contrast reference image for each measurement to mitigate variation in contrast [24]. For the calculated BB PSIR, the normal myocardial R1 value is needed, and for the calculated DB-PSIR the values for normal myocardium (R1m, R2m), as well as blood pool (R1b, R2b) are needed. The myocardium (endo- and epi-cardial borders) and blood pool were automatically segmented using an artificial intelligence (AI) approach using a convolutional neural network (CNN) facilitating a fully automated in-line display of the calculated PSIR LGE. AI segmentation was fully integrated using Gadgetron in-line AI to run on the scanner [28]. The median T1 and T2 in the segmented myocardium were used as estimates for the normal values.

### Measurements

LGE was performed using single dose (0.1 mmol/kg) of gadoterate meglumine (Dotarem; Guerbet, Villepinte, France) with acquisition approx. 10 min after contrast administration. A short axis (SAx) stack was first acquired using the BB PSIR MOCO protocol. A slice with MI was selected, followed immediately by acquiring the single slice with the DB PSIR MOCO and mSASHA protocols. The single SAx slice with MI was acquired for all imaging protocols within approximately 4 min of each other to minimize contrast variation. For CNR comparison, measurements were made on 10 subjects with chronic subendocardial MI, 3 with acute MI, and 1 subject with myocarditis.

Imaging was performed on a 1.5 T CMR scanner (MAGNETOM Aera, Siemens Healthineers, Erlangen, Germany). All patient scans were conducted at the Royal Free Hospital, London, UK or Hammersmith Hospital, London, UK. Ethical approvals obtained included prospective (London Hampstead Research Ethics Committee, reference 19/LO/1561) with written informed consent, and retrospective (East Midlands—18/EM/0341 Leicester Central Research Ethics Committee and East of England—Cambridge Central Research Ethics Committee, reference 21/EE/0037) use of anonymized CMR images, including for technical development. For those patients undergoing clinical scanning and included using retrospective ethics, we had local information governance approval for the sharing and publication of anonymized imaging data. All data were de-identified for analysis at NIH with approval by the local Office of Human Subjects Research (Exemption Approval for collaboration #13156).

### CNR measurements

SNR was measured in MI, remote myocardium, and left ventricular (LV) blood pool. SNR measurement used manually drawn contours. The CNR between MI and remote zone was calculated as the ratio of the signal difference to standard deviation of remote, and CNR between MI and blood was similarly calculated. SNR measurements for the BB and DB MOCO PSIR were made using the SNR scaled image reconstruction approach [29].

It is difficult to directly measure the SNR and CNR of the synthetic images using an SNR scaled reconstruction due to the non-linear relationship of the T1 and T2 mapping estimates. In order to calculate the SNR and CNR of synthetic LGE images, a Monte-Carlo approach was used which directly implements Eqs. (3 and 4). Similar Monte-Carlo methods for evaluation of T1 error in SR based T1 mapping have been previously validated [30]. The T1 and T2 for the 3 regions were measured on the maps, and the SNR for the 3 regions was also measured in the raw anchor images (with 3 recovery beats). The mean and standard deviation of the BB and DB PSIR signal in the 3 regions was calculated from Eqs. (3 and 4) by repeated trials, where the equations used the measured values of T1, T2, and SNR, and imaging protocol. SNR estimates used 10,000 trials. Measured values for T1 and T2 are reported in the results.

## Results

There were 14 subjects (12 male; 61 ± 10 years) with scar: 10 chronic MI, 3 acute MI, and 1 myocarditis. Patient weight ranged from 58.6 to 123.8 kg.

**Fig. 2:**
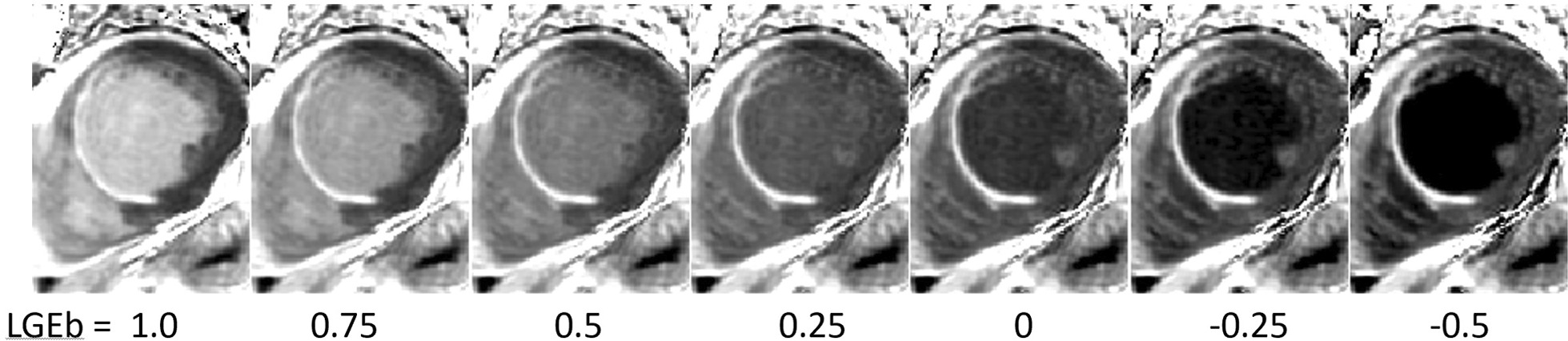
Patient with subendocardial myocardial infarction (MI) illustrating the synthetic phase sensitive inversion recovery (PSIR) late gadolinium enhancement (LGE) with varying blood suppression by adjusting the free parameter LGEb, defined in Eq. (4, 5)

An example of synthetic PSIR LGE for subject with subendocardial MI is show in Fig. 2. The DB PSIR signal calculated by Eq. (4) is calculated with varying values of blood suppression parameter LGEb as defined in Eq. (4), with bright blood on left (LGEb = 1), highly blood suppressed on right (LGEb = -0.5), and intermediate values of blood suppression in between.

**Fig. 3:**
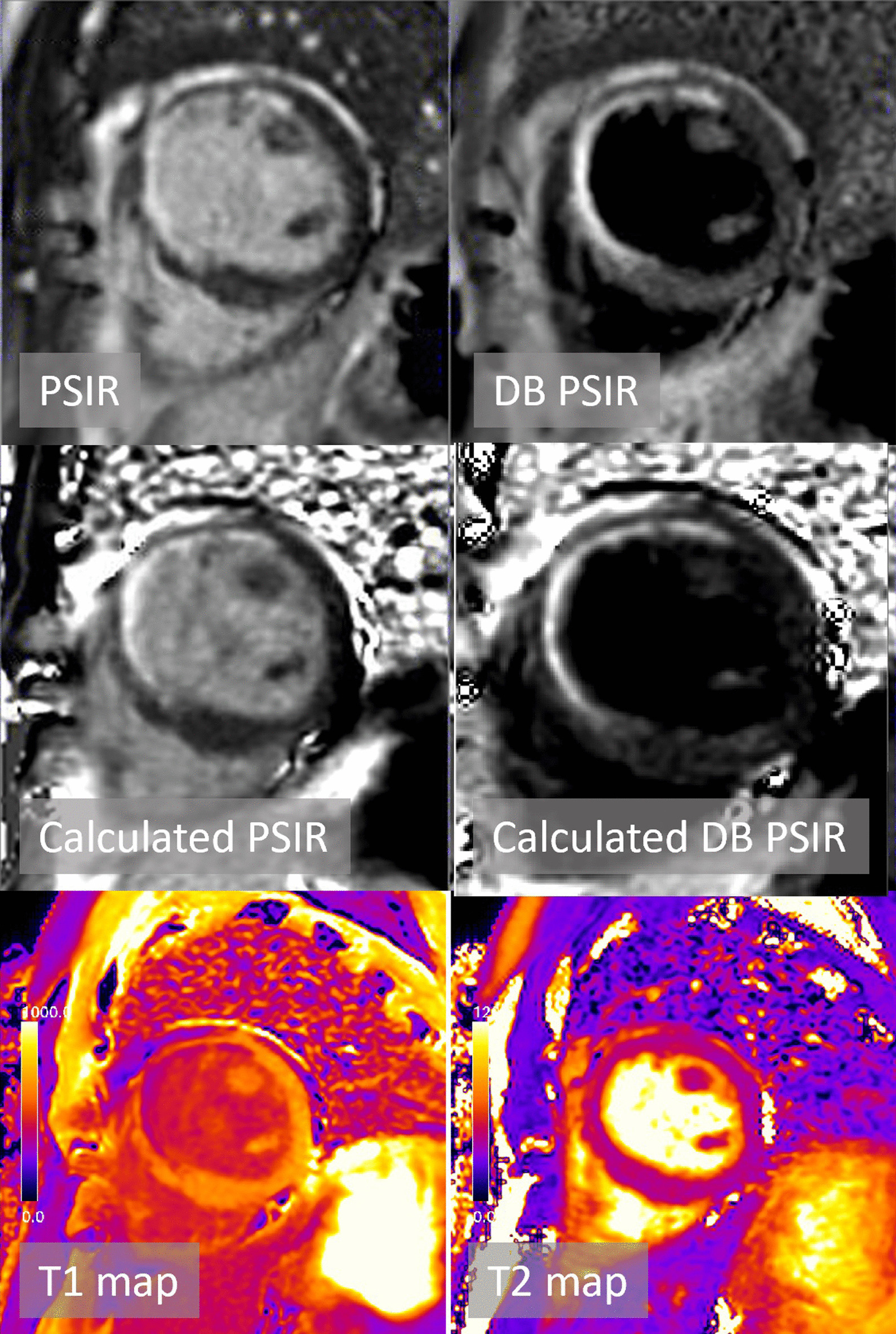
Patient with subendocardial MI with good MI-blood pool contrast readily seen in both bright blood (BB) and dark blood (DB) PSIR. Subendocardial MI is evident in the T1 map

**Fig. 4:**
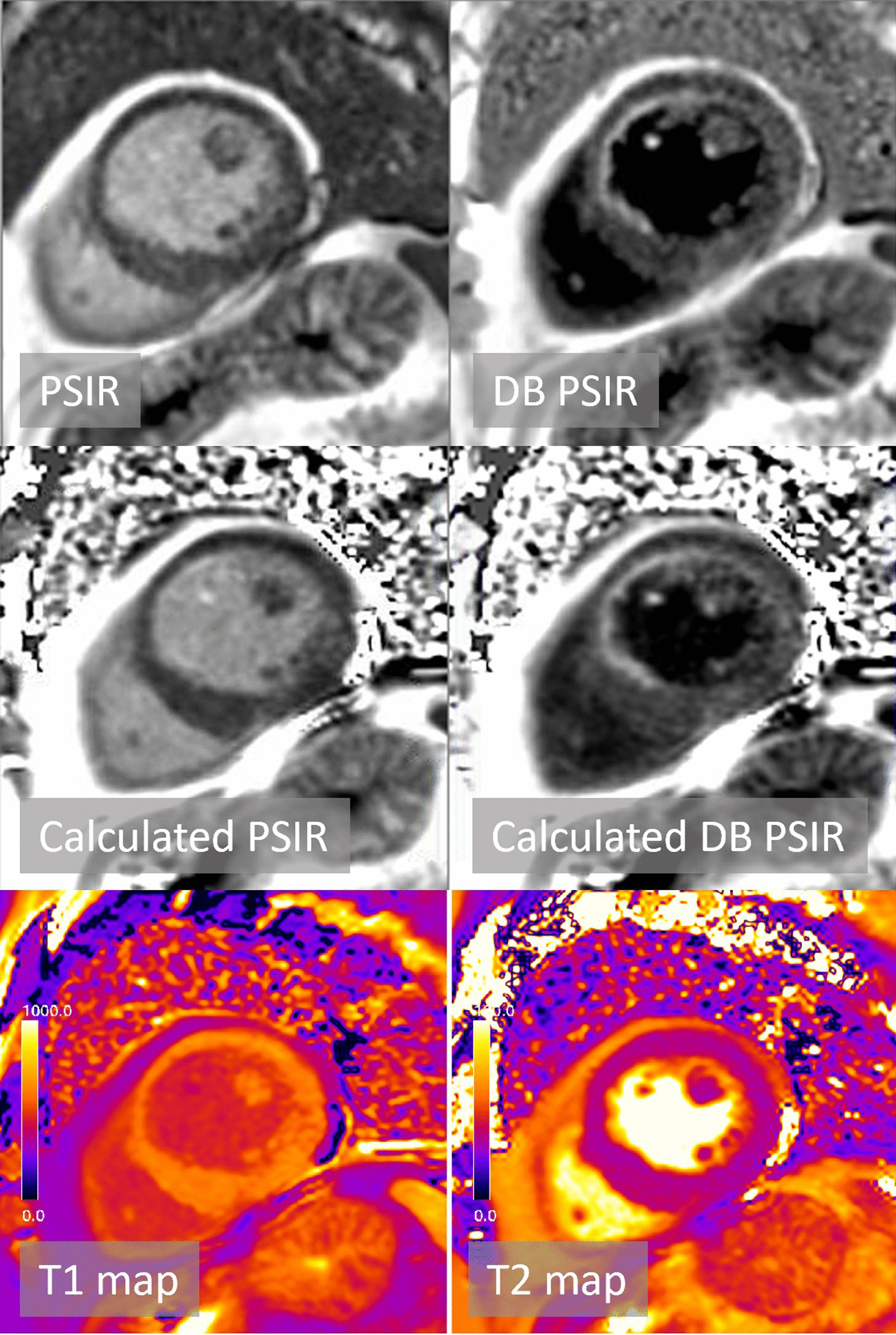
Patient with subendocardial MI with poor MI-blood pool contrast in BB PSIR but readily seen in DB PSIR. The T1 of MI is similar to adjacent blood pool making detection difficult in the T1-map

**Fig. 5:**
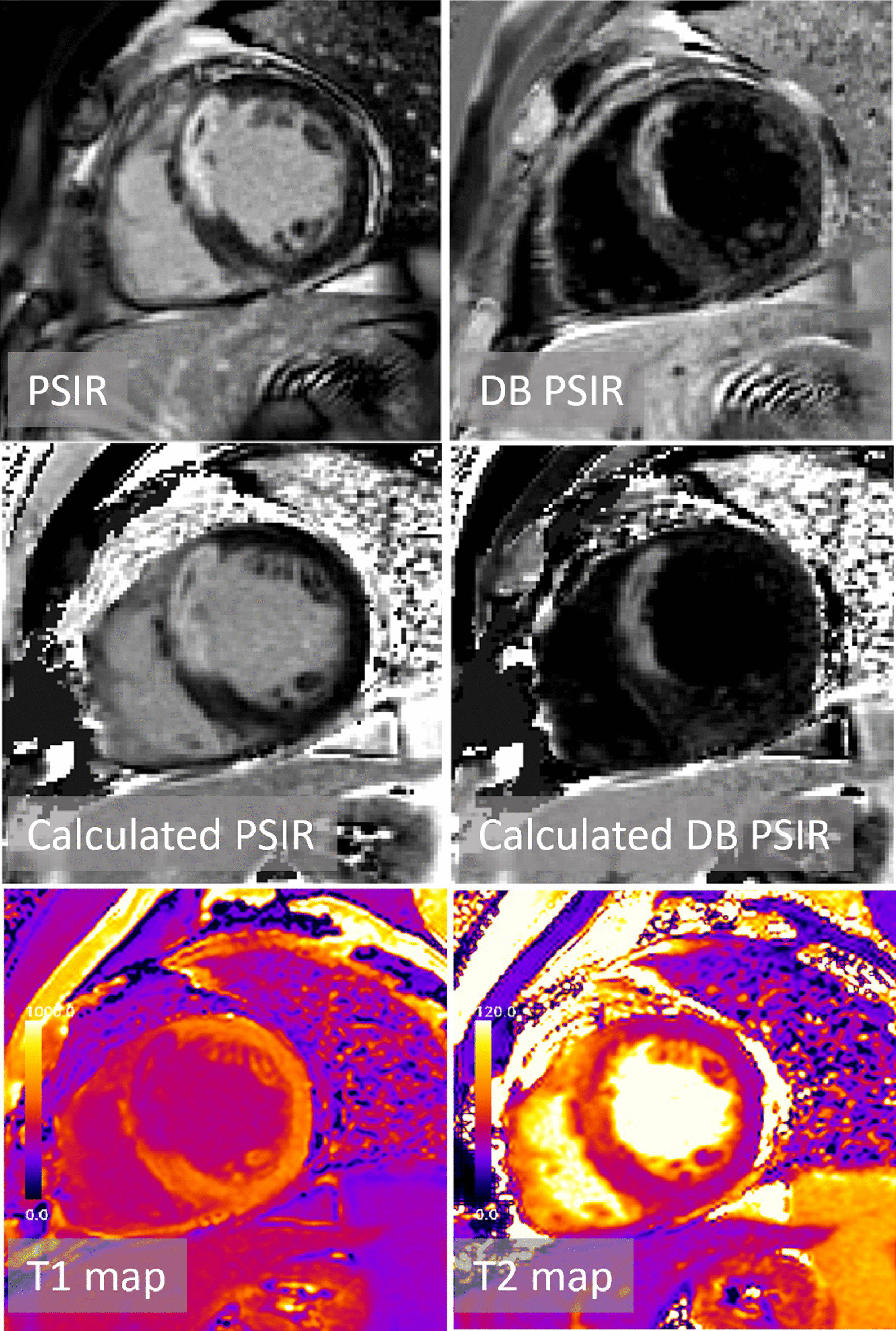
Patient with acute MI with elevated T2 and microvascular obstruction evident as dark core

**Fig. 6:**
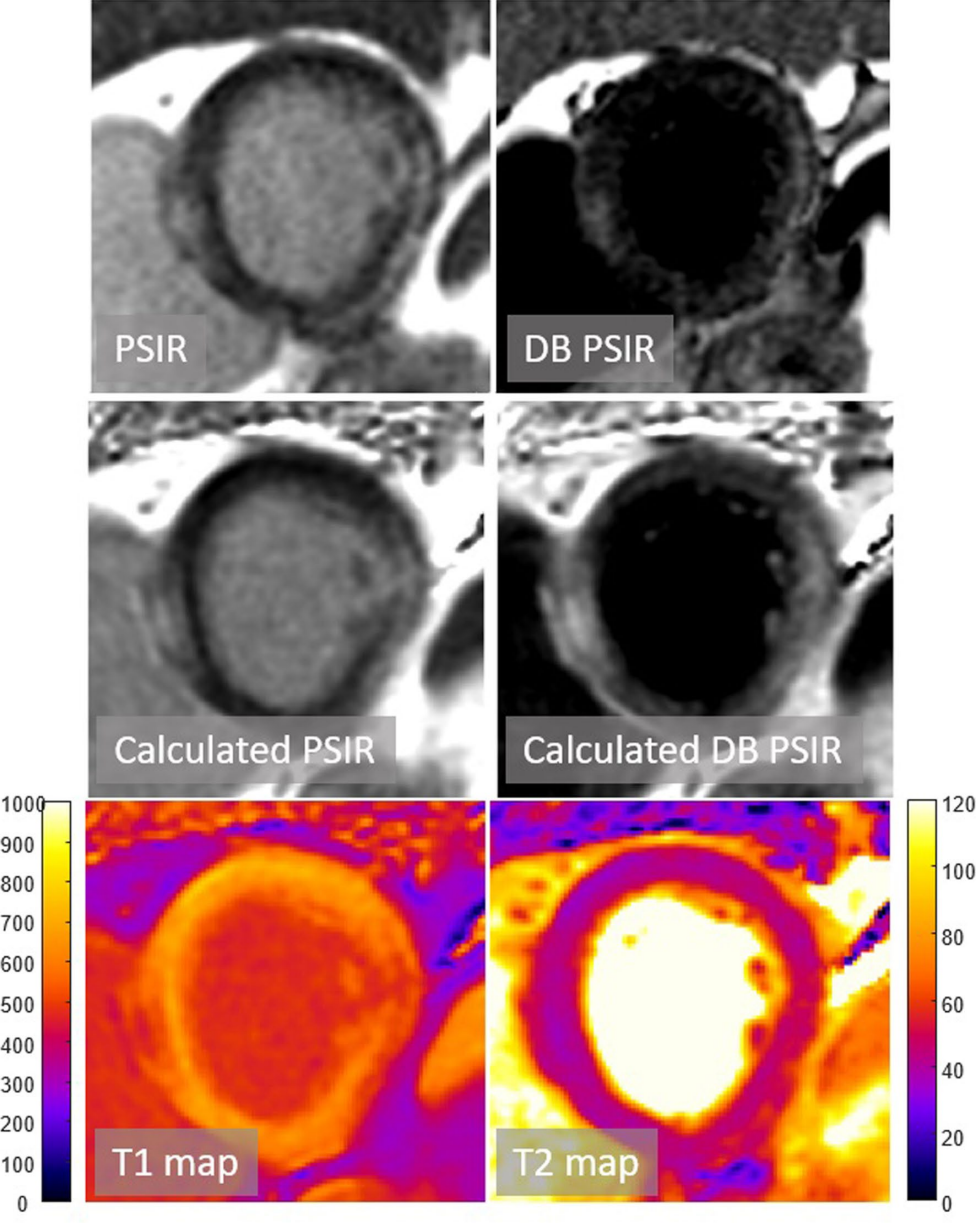
Patient with myocarditis and sub-epicardial LGE with mildly elevated T2

Typical example LGE images and maps are shown in Figs. 3 and 4 for two patients with subendocardial chronic MI. The quality of calculated PSIR LGE is comparable to conventionally acquired PSIR LGE. Figure 4 further demonstrates the benefit of DB LGE in a patient with poor contrast between the subendocardial MI and adjacent blood pool (Fig. 4). Cases with elevated T2 are shown for acute MI (Fig. 5) and myocarditis (Fig. 6).

**Fig. 7:**
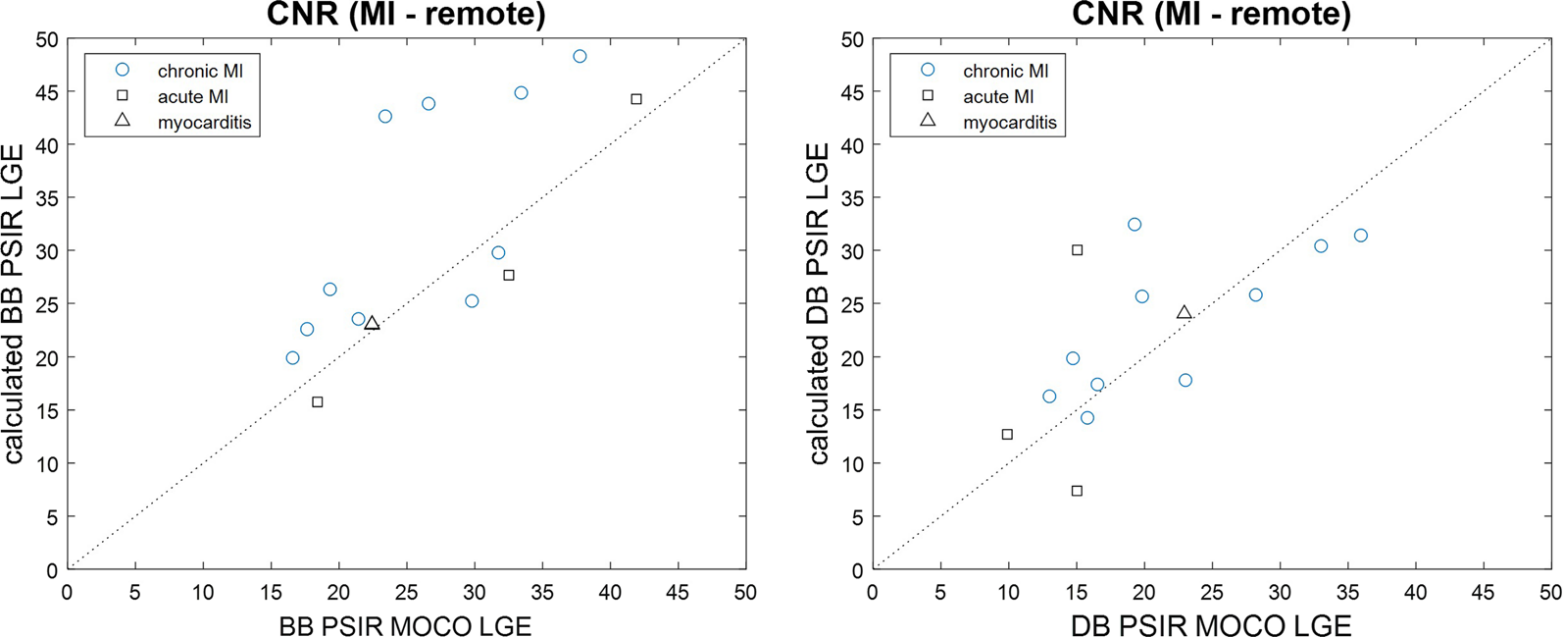
Comparison of measured contrast-to-noise ratio (CNR) between MI and normal myocardium for calculated PSIR vs MOCO PSIR with BB (Left) and DB (Right)

**Fig. 8:**
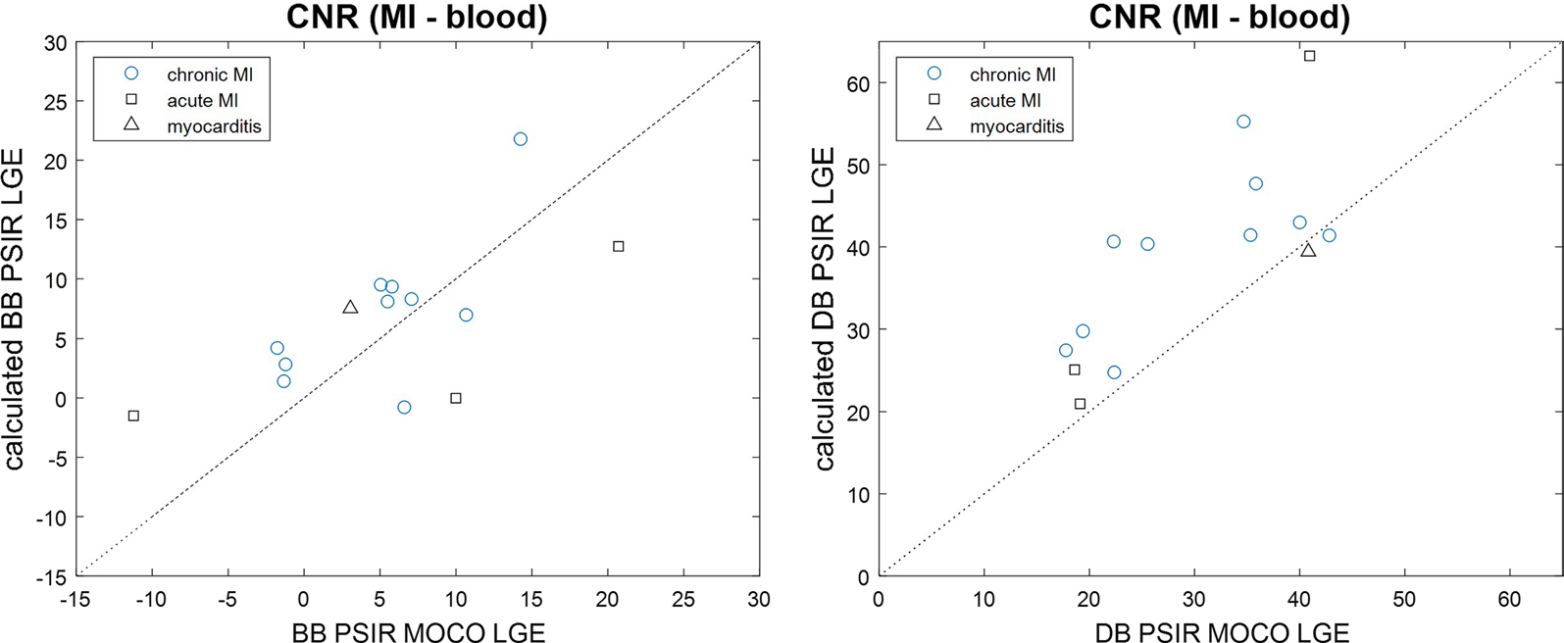
Comparison of measured CNR between MI and adjacent LV blood pool for calculated PSIR vs MOCO PSIR with BB (Left) and DB (Right)

The average CNR between scar and remote (Fig. 7) was 26.6 ± 7.7 and 20.2 ± 7.4 for conventional BB and DB PSIR LGE, and 31.3 ± 10.6 and 21.8 ± 7.6 for the calculated BB and DB PSIR LGE, respectively, in n = 14 patients. The CNR between MI and LV blood pool (Fig. 8) was 5.2 ± 6.5 and 29.7 ± 9.4 for conventional BB and DB PSIR LGE, and 6.5 ± 6.0 and 38.6 ± 11.6 for calculated BB and DB PSIR LGE, respectively, (n = 14). The correlation of the CNR between scar and remote for conventional vs calculated LGE had r^2^ = 0.52 for BB and r^2^ = 0.41 for DB. The correlation of the CNR between scar and blood for conventional vs calculated LGE had r^2^ = 0.41 for BB and r^2^ = 0.58 for DB.

In chronic MI (n = 10), measured values of T1 were 441 ± 68 ms in MI, 668 ± 60 in remote myocardium, and 466 ± 52 in the LV blood pool. Measured values of T2 were 43.8 ± 2.7 ms in MI, 42.4 ± 1.8 in remote myocardium, and 142.4 ± 7.6 in the LV blood pool. There was no statistical significance (p = 0.18) between T2 values in remote and MI territories. For patients with elevated T2, acute MI and myocarditis, the measured values of T1 were 392 ± 82 ms in MI, 608 ± 75 in remote myocardium, and 396 ± 84 in the LV blood pool. Measured values of T2 were 58.3 ± 3.2 ms in MI, 41.7 ± 2.4 in remote myocardium, and 141.8 ± 34.1 in the LV blood pool.

**Fig. 9:**
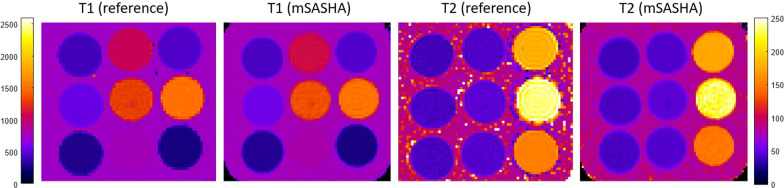
Phantom validation of proposed mSASHA using long TR scans as the standard of reference

Validation of T1 and T2 measurement accuracy was performed in a phantom with a set of vials with a range of T1 and T2 values (Fig. 9) to represent myocardium and blood with and without contrast. Measured values for mSASHA and the reference standard with long TR are listed in Table 2. T1 values with mSASHA were within 1% of the reference standard for all tubes. T2 values with mSASHA were within 1.5% for all gel tubes with myocardium like T2, and within 3.2% for liquid tubes with longer T2 typical of blood.

**Table 2:** Measured values of T1 and T2 in T1MES phantom

T1 (SE)	T1 (mSASHA)	T2 (SE)	T2 (mSASHA)
439	438	40.0	39.8
1111	1104	44.3	43.7
464	466	181.0	176.2
572	570	42.5	42.0
1361	1359	46.6	46.8
1527	1524	234.4	228.2
303	302	41.9	41.7
814	812	44.8	44.3
258	260	154.2	149.2

## Discussion

The proposed multi-parametric approach simultaneously provides T1 and T2 maps and BB and DB contrast LGE images for each 2D slice in 45 heartbeats during free-breathing. Compared to conventional imaging techniques with multiple separate scans for each of the desired images, the proposed all-in-one solution can both accelerate and simplify clinical imaging workflows. Acquiring the FB bright blood MOCO PSIR requires 16 heart beats per slice plus a TI scout to find the inversion time to null the normal myocardium. The currently used FB DB MOCO PSIR requires 32 heart beats per slice plus a T1-map used as “scout” to set-up the protocol. Thus, the proposed approach requiring 45 heart beats per slice is comparable in overall time with acquiring both bright and dark-blood PSIR MOCO LGE protocols (48 HBs per slice). Several additional minutes would be required to acquire a stack of T1 maps alone. A single protocol with a stack of slices covering the myocardium can be prescribed and no pause between slices is required since all measurements are preceded by saturation preparations. As the BB and DB LGE images are calculated from the T1 and T2 maps, TI and TE times do not require operator input, further simplifying workflow for the operator. Single-shot readouts can effectively reduce or eliminate respiratory artifacts during steady free-breathing, improving the patient experience compared to traditional breath-holds.

Assessment of global scar burden may be made with whole heart coverage, as well as global assessment of edema or diffuse fibrosis.

Another advantage of the proposed solution is the four output images are intrinsically co-registered, simplifying analysis and reporting. DB and BB LGE images can be directly compared as well as T1 and T2 maps, without potential slice mismatch between multiple separate scans. If combined with native mapping, extracellular volume (ECV) maps can also be generated and co-registered. The four output images can also be further used as different channel inputs in a deep learning model to improve classification and segmentation of myocardium. Further development may enable an AI based solution for automated analysis and reporting of relaxation time parameters and LGE enhancement.

Normal values for the myocardial T1 and T2 used for calculating the synthetic PSIR used the median value of segmented myocardium. The entire myocardium was segmented and the median was chosen for as an estimate of “normal” since it is less affected than the mean to regional abnormalities. However, PSIR is inherently insensitive to errors in the assumed normal value used for nulling over a broad range [31]. The calculated PSIR can be window-leveled to compensate an error in normal T1 just as the acquired PSIR can be retrospectively window-leveled to null the myocardium in situation when the TI is incorrect. For this purpose, it is acceptable to have some regional scar within the segmented myocardium.

In both conventional PSIR and calculated PSIR, bright blood LGE encounters challenging cases due to poor blood pool contrast with subendocardial MI. In these cases, both the conventional and calculated PSIR dark blood approached significantly improved the blood pool contrast facilitating detection. The measured blood pool contrast for the calculated DB PSIR LGE was slightly better than the acquired PSIR-T2 due mainly to differences in blood suppression criteria of the 2 implementations, but both achieve significantly improved contrast compared to their bright blood counterpart.

The formulation described by Eqs. (3 and 4) were implemented for this work since the signal is linearly related to gadolinium concentration rather than through an exponential recovery. Using the direct formulation (Eq. (4)) achieved a specified fixed level of blood signal in the calculated images, whereas the blood suppression for the conventional DB MOCO PSIR approach is variable [11]. As a result, the comparison of CNR between methods is expected to be somewhat variable. Both calculated and conventional DB methods achieve excellent blood pool contrast. The mean value of R1 was approximately 2.27, 1.49, and 2.15 Hz in MI, normal myocardium and blood pool, respectively, calculated from the measured values of T1 in these regions. The value of DB signal was set to − 0.5 Hz, somewhat arbitrarily, and provided excellent blood pool contrast. The IR-T2 sequence [11] with the acquired signal described by Eq. (2) selects sequence parameters (TD1, TD2, and TE) to set a specified time difference between null times for myocardium and blood which is a different criteria for blood suppression and results in more variable blood signal values.

A benefit of the multi-parametric SASHA approach is the ability to accurately measure both T1 and T2, i.e., without T1 confounding T2 or T1 confounding T2. Myocardial T1 and T2 are both influenced by water content and thus are generally both elevated in case of edema which may occur as a result of inflammatory response to acute injury. The interplay between T1 and T2 will also depend on the molecular content such as protein and extracellular matrix. Study of myocardial T1 and T2 has been difficult with many of the widely used techniques, thus the proposed method may provide new insights. The proposed method will also permit exploring the potential value of T2 in the presence of contrast.

Using the multi-parametric SASHA it is possible to accurately measure the T2 at short T1, without the confounding bias [32] due to T1-recovery. Values for T2 in the blood are significantly reduced with contrast. Native T2 for arterial blood is approx. 250 ms, whereas the mean T2 measured approx. 10 min following 0.1 mmol/kg gadoterate meglumine was approx. 142 ms. This corresponds to a contrast concentration of 0.52 mmol/L, assuming relaxivity r2 = 5.85 (sec^−1^ mmol^−1^) [33]. Measured T2 values in subendocardial MI were slightly elevated due to contamination from adjacent blood pool, but did not reach statistical significance.

Myocardial T2 is also reduced by gadolinium, albeit to a lesser extent than the blood pool. Therefore, post contrast T2 measurement, although accurate using mSASHA, is not recommended as a substitute for native T2 for the detection of global abnormalities such as edema.

Blood pool inhomogeneity is evident in the calculated BB PSIR LGE in Fig. 3. It is possible that this is due to flow sensitivity of the T2 preparation, and that it might be more homogenous by further protocol optimization by addition of T2p images with shorter TE.

## Limitations

There are limitations to our study. First, the proposed method was tested at 1.5 T and using a single contrast agent at a single dose at a single imaging center. Evaluation at other doses and field strengths in a multi-center study is warranted. Second, while the image quality was evaluated against conventional PSIR with CNR measurements, diagnostic performance of calculated PSIR LGE images was not compared. The assumption that the R1 is directly proportional to contrast concentration was not verified here for MI and relies on an assumption of fast exchange that is believed to be reasonable at typical contrast doses administered. Unlike the calculated LGE based on measured R1, the exponential inversion recovery signal is highly non-linear.

The current comparison of quality is based on the measured CNRs and the overall visual appearance. A comparison of MI size was not performed in this study. A more comprehensive comparison of LGE quality would require volumetric coverage which was not undertaken here due to the desire of having multiple sequences acquired within close proximity in time. A more complete comparison of infarct size as well as clinical utility is suggested as a next step.

Although there is growing acceptance of the use for quantitative mapping for detection of diffuse disease, patients with diffuse disease were not included in this study which focused on extending mapping to include LGE. The mSASHA approach to mapping may be applied for both native mapping as well as contrast to enable calculation of ECV. The proposed method is limited to tissue characterization, and overall cardiac assessment will include measurements of volume and flow as appropriate.

**Fig. 10:**
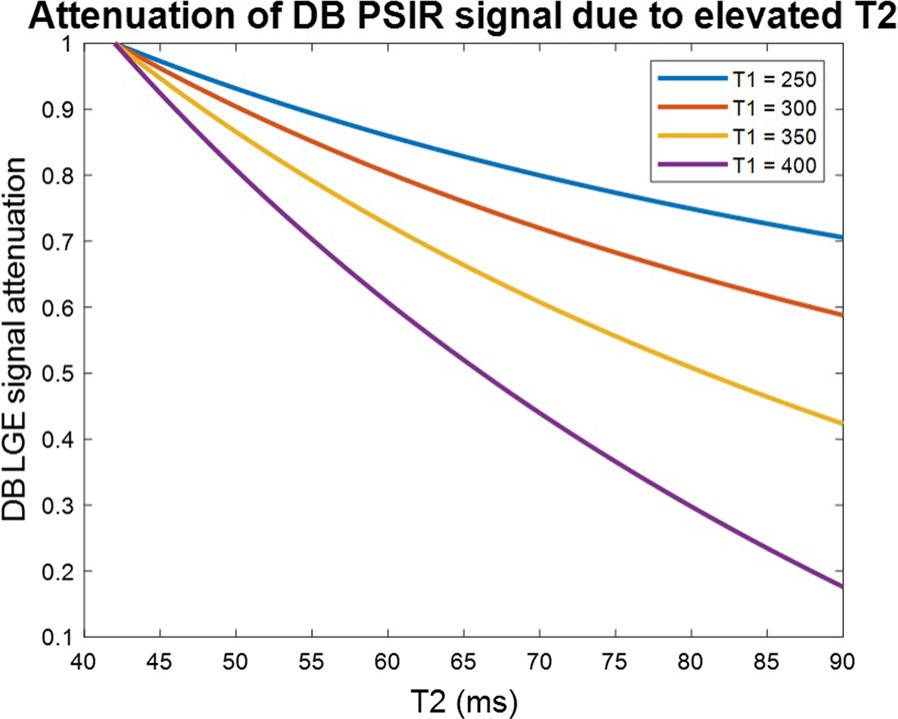
Attenuation of DB PSIR LGE signal in cases of elevated myocardial T2 arising from edema shown for varying T1 of edematous region

The DB PSIR signal will be attenuated in cases of edema that arises in cases such as acute MI or acute myocarditis. Although, the signal was not significantly attenuated in the few cases reported here, it may be significant for cases with more severe T2 elevation. Attenuation of the DB PSIR signal calculated in Eq. (4) depends on several variables such as degree of both T1 and T2 elevation as well as the blood T1 and T2. A plot of DB PSIR signal attenuation is shown in Fig. 10 for 1 set of parameters (remote myocardial T1/T2 = 600/42 ms, blood T1/T2 = 400/150 ms) and varying T1 and T2 for the region of edematous myocardium (i.e., acute MI). Cases with very high T2 elevation are likely to have shorter T1, thus limiting the signal attenuation that will be encountered. In such scenarios, the user will observe significant elevation in myocardial T2. The signal attenuation also depends on the degree of blood suppression (LGEb) and will be less with less suppression (i.e., gray blood).

## Conclusion

This study utilized a single free-breathing mSASHA acquisition to provide simultaneous T1 and T2 maps and calculated BB and DB PSIR LGE for improved visualization of subendocardial MI. The proposed method provides a practical approach for comprehensive tissue characterization which is readily integrated into the clinical workflow. All images are spatially co-registered improving the confidence for assessment of focal disease and simplifying analysis.

## Data Availability

The raw data that support the findings of this study are available from the corresponding author upon reasonable request subject to restriction on use by the Office of Human Subjects Research. Raw data requires reconstruction processing.

## Abbreviations

AI: Artificial intelligence
BB: Bright blood
BH: Breath-hold
CMR: Cardiovascular magnetic resonance
CNN: Convolutional neural network
CNR: Contrast-to-noise ratio
DB: Dark blood
ECV: Extracellular volume
FB: Free-breathing
IR: Inversion recovery
LGE: Late gadolinium enhancement
LV: Left ventricle/left ventricular
MI: Myocardial infarction
MOCO: Motion correction
mSASHA: Multi-parametric SASHA
PSIR: Phase sensitive inversion recovery
SASHA: Saturation recovery single-shot acquisition
SAx: Short axis
SNR: Signal-to-noise ratio
SR: Saturation recovery
T2p: T2 preparation
TE: Echo time
TI: Inversion time
